# Forecasting of Patient-Specific Kidney Transplant Function With a Sequence-to-Sequence Deep Learning Model

**DOI:** 10.1001/jamanetworkopen.2021.41617

**Published:** 2021-12-30

**Authors:** Elisabet Van Loon, Wanqiu Zhang, Maarten Coemans, Maarten De Vos, Marie-Paule Emonds, Irina Scheffner, Wilfried Gwinner, Dirk Kuypers, Aleksandar Senev, Claire Tinel, Amaryllis H. Van Craenenbroeck, Bart De Moor, Maarten Naesens

**Affiliations:** 1 Department of Microbiology, Immunology and Transplantation, Nephrology and Kidney Transplantation Research Group, KU Leuven, Leuven, Belgium; 2 Department of Nephrology and Kidney Transplantation, University Hospitals Leuven, Leuven, Belgium; 3 ESAT STADIUS Center for Dynamical Systems, Signal Processing and Data Analytics, KU Leuven, Leuven, Belgium; 4 Department of Development and Regeneration, KU Leuven, Leuven, Belgium; 5 Histocompatibility and Immunogenetic Laboratory, Red Cross Flanders, Mechelen, Belgium; 6 Department of Nephrology, Hannover Medical School, Hannover, Germany

## Abstract

**Importance:**

Like other clinical biomarkers, trajectories of estimated glomerular filtration rate (eGFR) after kidney transplant are characterized by intra-individual variability. These fluctuations hamper the distinction between alarming graft functional deterioration or harmless fluctuation within the patient-specific expected reference range of eGFR.

**Objective:**

To determine whether a deep learning model could accurately predict the patient-specific expected reference range of eGFR after kidney transplant.

**Design, Setting, and Participants:**

A multicenter diagnostic study consisted of a derivation cohort of 933 patients who received a kidney transplant between 2004 and 2013 with 100 867 eGFR measurements from University Hospitals Leuven, Belgium, and 2 independent test cohorts: with 39 999 eGFR measurements from 1 170 patients, 1 from University Hospitals Leuven, Belgium, receiving transplants between 2013 and 2018 and 1 from Hannover Medical School, Germany, receiving transplants between 2003 and 2007. Patients receiving a single kidney transplant, with consecutive eGFR measurements were included. Data were analyzed from February 2019 to April 2021.

**Exposures:**

In the derivation cohort 100 867 eGFR measurements were available for analysis and 39 999 eGFR measurements from the independent test cohorts.

**Main Outcomes and Measures:**

A sequence-to-sequence model was developed for prediction of a patient-specific expected range of eGFR, based on previous eGFR values. The primary outcome was the performance of the deep learning sequence-to-sequence model in the 2 independent cohorts.

**Results:**

In this diagnostic study, a total of 933 patients in the training sets (mean [SD] age, 53.5 [13.3] years; 570 male [61.1%]) and 1170 patients in the independent test sets (cohort 1 [n = 621]: mean [SD] age, 58.5 [12.1] years; 400 male [64.4%]; cohort 2 [n = 549]: mean [SD] age, 50.1 [13.0] years; 316 male [57.6%]) who received a single kidney transplant most frequently from deceased donors, the sequence-to-sequence models accurately predicted future patient-specific eGFR trajectories within the first 3 months after transplant, based on the previous graft eGFR values (root mean square error, 6.4-8.9 mL/min/1.73 m^2^). The sequence-to-sequence model predictions outperformed the more conventional autoregressive integrated moving average prediction model, at all input/output number of eGFR values.

**Conclusions and Relevance:**

In this diagnostic study, a sequence-to-sequence deep learning model was developed and validated for individual forecasting of kidney transplant function. The patient-specific sequence predictions could be used in clinical practice to guide physicians on deviations from the expected intra-individual variability, rather than relating the individual results to the reference range of the healthy population.

## Key Points

**Question:**

Can a deep learning model accurately predict patient-specific estimated glomerular filtration rate (eGFR) ranges?

**Findings:**

In this diagnostic study in a derivation cohort of 933 single kidney transplant recipients with 100 867 eGFR values and validation cohort of 1170 single kidney transplant recipients with 39 999 eGFR values, a sequence-to-sequence model was able to accurately predict patient-specific eGFR ranges within the first 3 months after transplant, based on the grafts’ previous eGFR values.

**Meaning:**

Findings of this diagnostic study suggest that the patient-specific sequence predictions could be used in clinical practice to guide physicians to identify deviations from the expected intra-individual variability.

## Teaser

This diagnostic study assesses whether a deep learning model could accurately predict the patient-specific expected reference range of estimated glomerular filtration rate after kidney transplant.

## Introduction

There is a wide variability in kidney function, calculated as the estimated glomerular filtration rate (eGFR) between transplant recipients, depending on quality of the donor organ but also on recipient-related factors. However, also within patients, the eGFR fluctuates, as is the case with all clinical biomarkers that are monitored over time. This intra-individual variability can range from fluctuations explained by technical or analytical variability in the measurement^1,2,3^ and biological variability associated with hemodynamic changes, dietary intake, muscle mass, physical exertion, among others to actual damage from acute events such as ischemia-reperfusion and specific posttransplant injury processes such as transplant rejection.^4,5^

Given the wide between-patient variability, the static evaluation of eGFR is less informative for clinical decision-making. Therefore, experienced clinicians take the patient-specific fluctuations of eGFR into account. Variability in eGFR values is interpreted as truly alerting, prompting action, such as performing a kidney transplant biopsy, or as quiescent, falling in a range that can be considered normal for this patient. The inherent fluctuating nature of eGFR often leaves clinicians in doubt on whether a decrease in eGFR can still be considered in the reference range of fluctuation or whether it should alert them to prompt a biopsy. In patients with chronic disease, many parameters are systematically out of the ranges as defined for healthy individuals, including the eGFR values of transplanted kidneys. This maladapted reference range complicates the use of standardized actions and leads to less reproducible clinical estimation of indications for action and could be the reason that alert systems for acute kidney injury often fail in realizing hard end points,^6,7,8^ because not taking into account the individual day-to-day variability may lead to generation of numerous less relevant alerts, inducing alert fatigue.^9^ Tools that translate time-dependent intra-individual eGFR fluctuations into a useful patient-specific guide for clinical decision-making may have utility. Therefore, in this study we aimed to forecast future eGFR sequences of each individual graft based on the input of previous eGFR values with variable input lengths (previous eGFR trajectories) and output lengths (forecasted eGFR sequences). The accuracy of these predictions was evaluated by comparison of the forecasted eGFR sequences with the real observed eGFR values. The predicted eGFR sequences could be considered as a patient-specific expected range, against which new measured values could be benchmarked to assess deviations from the predicted range, which could then prompt patient-specific alarms in clinical practice.

## Methods

### Study Population and Data Collection

For the derivation cohort, all consecutive adult recipients of a single kidney transplant at the University Hospitals Leuven between March 2004 and February 2013 were eligible. All transplants were performed with compatible complement-dependent cytotoxicity crossmatches. The clinical data were prospectively collected during routine clinical follow-up in electronic medical records, which were used for clinical patient management and directly linked to the SAS database (SAS Institute). This study followed the Standards for Reporting of Diagnostic Accuracy (STARD) reporting guideline for diagnostic studies.

For independent validation, we assessed the model performance in 2 separate test cohorts. For inclusion, eGFR values had to be available from the first 5 days after transplant. The first test cohort consisted of all 621 patients who received a transplant with a single kidney transplant between March 2013 and 2018 at the University Hospitals Leuven; the second test cohort consisted of 549 patients who received a transplant between 2003 and 2007 at Hannover Medical School. Data were analyzed from February 2019 to April 2021. This study was approved by the ethics committees of the University Hospitals Leuven and Hannover Medical School. All patients provided written informed consent.

### Determination of Kidney Function, Handling of Missing Values, and Biopsy Policy

Kidney allograft function was assessed by the glomerular filtration rate estimated by the 4-variable Modification of Diet in Renal Disease Study equation.^10^ The eGFR values were assessed routinely during hospitalizations and outpatient clinic visits with variable intervals. On days when no eGFR values were available (eFigure 1 in the Supplement), we used duplication as imputation method, meaning the last observation was carried forward for the input eGFR sequences.

Patients were followed up until graft failure (return to dialysis or preemptive retransplant) or until the date of data extraction (August 31, 2017). Decisions to perform indication biopsies were based on graft dysfunction, as judged by the treating clinicians. We opted to focus primarily on the eGFR values in the first 3 months after transplant given the availability of frequent sampling and the higher rate of fluctuations in this early posttransplant period, with a greater need for a patient-specific eGFR reference.

### Statistical Analysis

Characteristics of patients are described by means and SDs for continuous variables or median and IQR, as appropriate, and frequencies and percentages for categorical variables. We first applied an autoregressive integrated moving average (ARIMA), a traditional forecasting model, in a multistep out-of-sample forecast, on the 36 451 eGFR values collected in the first 3 months of the derivation cohort. More information on the ARIMA model can be found in eMethods in the Supplement. The performance of this conventional model was then used as benchmark.

Next, we applied a deep learning model for eGFR prediction.^11^ As detailed in eMethods in the Supplement, we opted for a sequence-to-sequence model. This model is a recurrent neural network–based model with an encoder-decoder architecture, which takes a sequence of values as input and outputs a sequence of predicted values. In the setting of this study, this model starts from a sequence of previous eGFR values to then predict future eGFR values for each individual graft (eFigure 2A in the Supplement). For this process, we used all eGFR data available in the first 3 months after transplant of the derivation cohort. Models with different input and output length were trained and evaluated. More details on the processing and concept of this model can be found in eMethods in the Supplement.

### Model Accuracy

The validation process consisted of 2 steps, as outlined in eFigure 2B in the Supplement, first by internal cross-validation on the derivation cohort and second by validating the locked models in the independent test cohorts. Details on the validation process can be found in eMethods in the Supplement. The model accuracy was assessed by comparing each predicted eGFR value from the output sequence and the real measured eGFR on the same day. The root mean square error reflects the difference in predicted vs measured eGFR value in mL/min/1.73 m^2^. The final sequence-to-sequence model performance was obtained by averaging the results of the 5-fold-trained models. For testing the accuracy of the sequence-to-sequence model beyond 3 months posttransplant, we trained new models for longer term input and output sequences of the derivation cohort (see eMethods in the Supplement).

### Model Performance for Prediction of Indication Biopsies

Mimicking current clinical decision-making, we assessed whether eGFR at the time of kidney transplant biopsies for graft dysfunction (indication biopsies) in the first 3 months after transplant deviated negatively from the predicted eGFR values from the sequence-to-sequence model. For this process, we excluded the 4 last eGFR values before an indication biopsy from the input sequence and compared the eGFR predictions from the models with the actual measured eGFR value on the biopsy dates. The models with the closest input length to the available input eGFR values of the patients (excluding the 4 last eGFR values before a biopsy) were used. The range of the predicted eGFR from these models was then represented by median and IQR. Next, we identified whether actual measured eGFR values fell inside or out of the IQR of the predicted eGFR and defined ‘worsening graft function’ when the actual eGFR on the day of the biopsy was below the lower quartile of the eGFR predictions. Biopsies performed within the first 5 days after transplant could not be considered for this approach.

We used Keras-2.2.4 TensorFlow version 1.13.1 in Python software version 3.6.10 (Python Software Foundation) for model programming. GraphPad Prism, version 8 (GraphPad Software) was used for data presentation.

## Results

### Study Population Characteristics

Overall, 2104 individual transplants were included in the study, divided into a derivation cohort (N = 933; mean [SD] age, 53.5 [13.3] years; 570 male [61.1%]) and 2 independent test cohorts (cohort 1: N = 621; mean [SD] age, 58.5 [12.1] years; 400 male [64.4%]; and cohort 2: N = 549; mean [SD] age, 50.1 [13.0] years; 316 male [57.6%]). Median follow-up time was 7.8 years (IQR, 5.4-10.4 years) in the derivation cohort, and 3.9 years (IQR, 2.6-5.2 years) in 1 independent test cohort and 10.9 years (IQR, 6.4-12.7 years) in the other independent test cohort. The characteristics of the cohorts are listed in Table 1. In brief, the cohorts consisted of 85.5% of first kidney transplant procedures, mostly (77.1%) from donors after brain death, with a male predominance in recipients and donors, and most (85.5%) receiving calcineurin-based immunosuppressive regimens. eFigure 3 in the Supplement depicts the between-patient variability in eGFR trajectories in the data set as well as within-patient variability . The widest variability can be noted in the first months after transplant.

**Table 1.** Characteristics of the Derivation and the Test Cohorts

Characteristic	Derivation cohort (n = 933)	Test cohort 1 (n = 621)	Test cohort 2 (n = 549)
Recipient characteristics at transplant			
Age, mean (SD), y	53.5 (13.3)	58.5 (12.1)	50.1 (13.0)
Sex, No. (%)			
Male	570 (61.1)	400 (64.4)	316 (57.6)
Female	363 (38.9)	221 (35.6)	233 (42.4)
Repeated transplant, No. (%)	140 (15.0)	92 (14.8)	73 (13.3)
Donor characteristics			
Age, mean (SD), y	47.7 (14.9)	50.0 (14.3)^a^	49.4 (14.8)
Sex, No. (%)			
Male	502 (53.8)	337 (54.3)^a^	299 (54.5)
Female	431 (46.2)	268 (43.2) ^a^	250 (45.5)
Type of donor, No. (%)			
Living donors	53 (5.7)	59 (9.5)	87 (15.9)
Donation after brain death	728 (78.0)	432 (69.6)	462 (84.2)
Donation after cardiac death	152 (16.3)	130 (20.9)	0
Transplant characteristic			
Cold ischemia time, mean (SD), h	14.2 (5.7)	11.7 (6.5)	13.9 (7.3)
No. (%) with initial immunosuppression regimen: CNI-MPA-CS	869 (93.1)	587 (94.5)^a^	349 (63.6)
Overall graft survival^b^ (%), y			
1	94.2	94.0	94.0
3	87.9	86.4	84.6
5	80.6	80.4	81.4
Death-censored graft survival^c^ (%), y			
1	95.7	95.5	95.4
3	92.8	93.4	89.9
5	89.4	93.4	87.9
eGFR measurement in the first 3 mo posttransplant			
No.	36 451	23 742	16 257
Mean (SD)	39.2 (11.8)	38.4 (14.7)	29.6 (13.1)

Abbreviations: CNI, calcineurin inhibitors; CS, corticosteroids; MPA, mycophenolic acid.

^a^ Missing data (N = 22 for donor age; N = 16 for donor sex; N = 37 for immunosuppressive regimen).

^b^ Overall graft survival: composite of graft failure and recipient death.

^c^ Death-censored graft survival: graft failure censored at time of recipient death with a functioning graft.

### ARIMA Modeling and Performance

We first applied the conventional forecasting model, ARIMA, with different input and output lengths to forecast patient-specific eGFR values in the derivation cohort applied in the first 3 months after transplant. This ARIMA model demonstrated potential for forecasting eGFR sequences, albeit with remaining inaccuracies compared with the actual eGFR values between 7.5 and 11.4 mL/min/1.73 m^2^ (Table 2).

**Table 2.** Performance of ARIMA and Sequence-to-Sequence Models in the Derivation Cohort^a^

Sequence	RMSE (mL/min/1.73 m^2^)	
	Derivation cohort	
	ARIMA	Sequence-to-sequence
IN: 5/OUT: 5	11.38	6.40
IN: 5/OUT: 15	9.25	6.92
IN: 30/OUT: 30	7.62	6.59
IN: 45/OUT: 45	7.48	6.94
IN: 90/OUT: 90	10.20	8.90

Abbreviations: ARIMA, auto-regressive integrated moving average; IN, input sequence of eGFR values; OUT, requested output length in days of eGFR predictions; RMSE, root mean square error.

^a^ Both an ARIMA model and the sequence-to-sequence model were applied on the derivation cohort. A 5-fold cross-validation was used for the sequence-to-sequence model and the mean of the 5-fold error rates was used for evaluation.

### Sequence-to-Sequence Model Development and Performance

**Figure 1.**
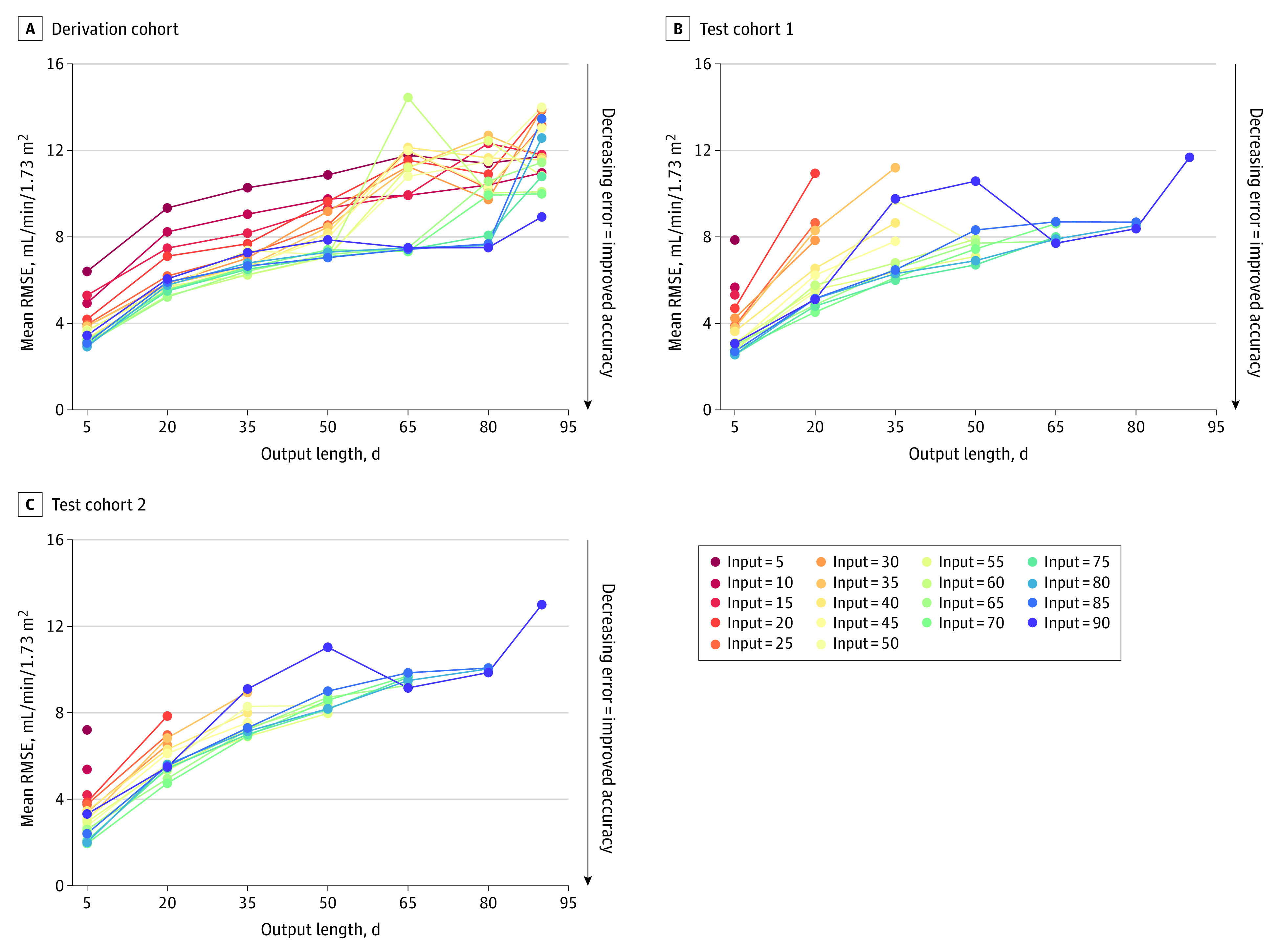
Performance of the Sequence-to-Sequence Models in the Derivation and Test Cohorts A, The accuracy of the sequence-to-sequence models is assessed by comparing the real observed eGFR values with the predicted eGFR values from the model. The difference, or root mean square error, is expressed in mL/min/1.73 m^2^. The lower the error, the better the prediction performance of the model. In the derivation cohort each forecasting result has 1 candidate prediction from each fold-trained model. The final performance is the average over the root mean square error of the 5 folds trained models on all patients. B, Performance of the sequence-to-sequence models in test cohort 1. C, Performance of the sequence-to-sequence models in test cohort 2. In the test cohorts, each forecasting result had 5 candidate predictions, which were used to calculate the mean RMSE (mean candidate eGFR vs observed eGFR). eGFR: estimated glomerular filtration rate; RMSE, root mean square error.

**Figure 2.**
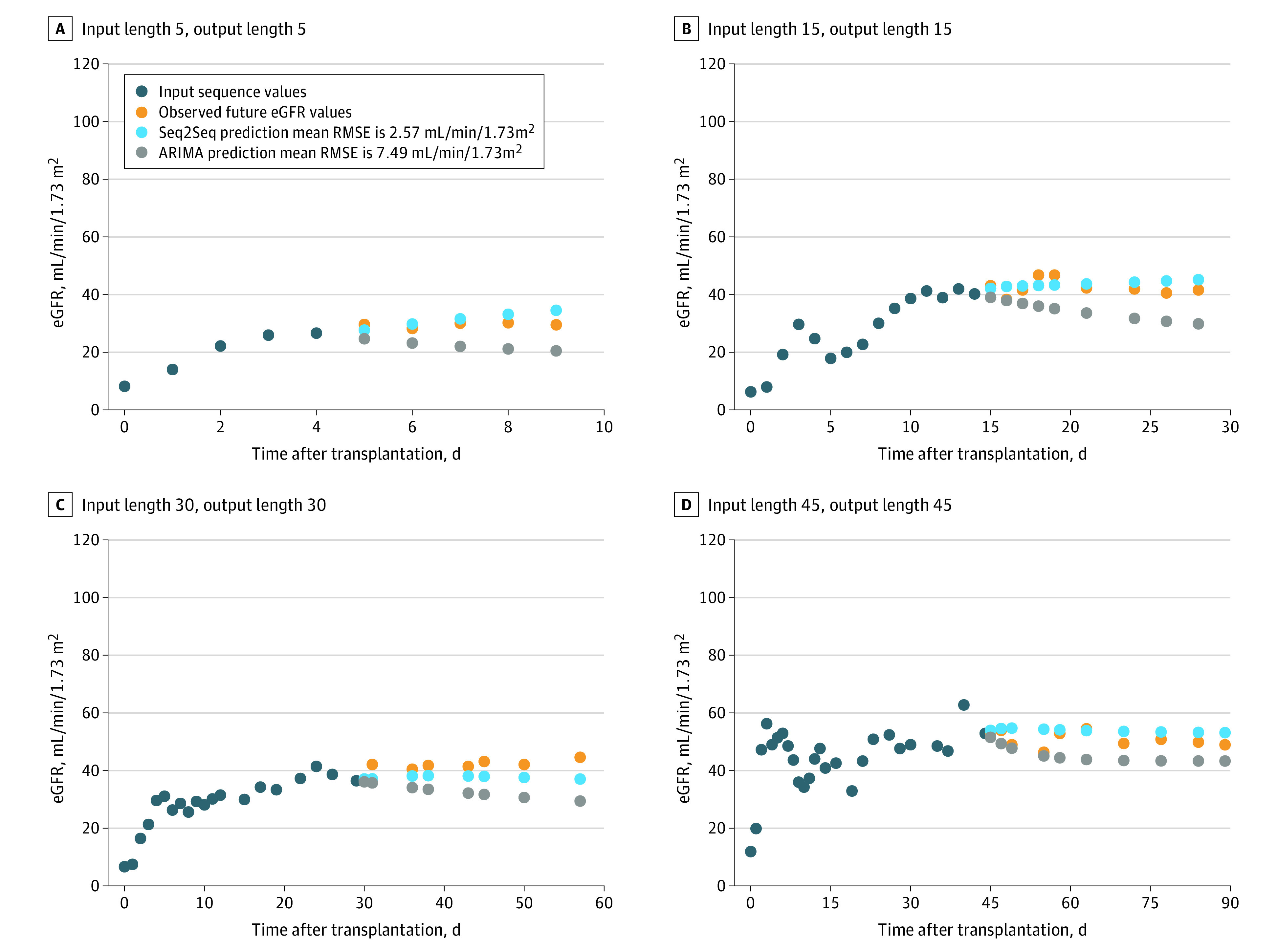
Graphical Illustration of the Patient-Specific Prediction of Future eGFR Trajectories Using the Sequence-to-Sequence Models, and Comparison With ARIMA Modeling, for 4 Randomly Selected Exemplary Cases A, Input length 5, output length 5; B, Input length 15, output length 15; C, Input length 30, output length 30. D, Input length 45, output length 45. ARIMA indicates auto-regressive integrated moving average; eGFR: estimated glomerular filtration rate; RMSE, root mean square error.

Next, we developed a deep learning sequence-to-sequence model, hypothesizing that this model could outperform conventional models in predicting eGFR sequences. We built sequence-to-sequence models on the 36451 eGFR values collected in the first 3 months of the derivation cohort, with varying input sequence and output sequence lengths. The root mean square error, expressed in mL/min/1.73 m^2^, between the output sequence predictions from the models and the actual measured eGFR values is depicted in Figure 1. The model predictions were more accurate with longer supplied input length (longer time frame of previous eGFR values) and shorter demanded output lengths (shorter time frame of future predicted eGFR values). Compared with the performance of the ARIMA model applied on the same data set, the sequence-to-sequence model outperformed the ARIMA model for all the provided input and requested output lengths, with inaccuracies compared with the actual eGFR values between 6.4 and 8.9 mL/min/1.73 m^2^ (Table 2). The greater predictive performance of the sequence-to-sequence algorithm compared to the measured eGFR values and to the ARIMA model was visually confirmed in randomly selected exemplary cases (Figure 2).

### Prediction of eGFR Evolution at Time of Indication Biopsies

**Figure 3.**
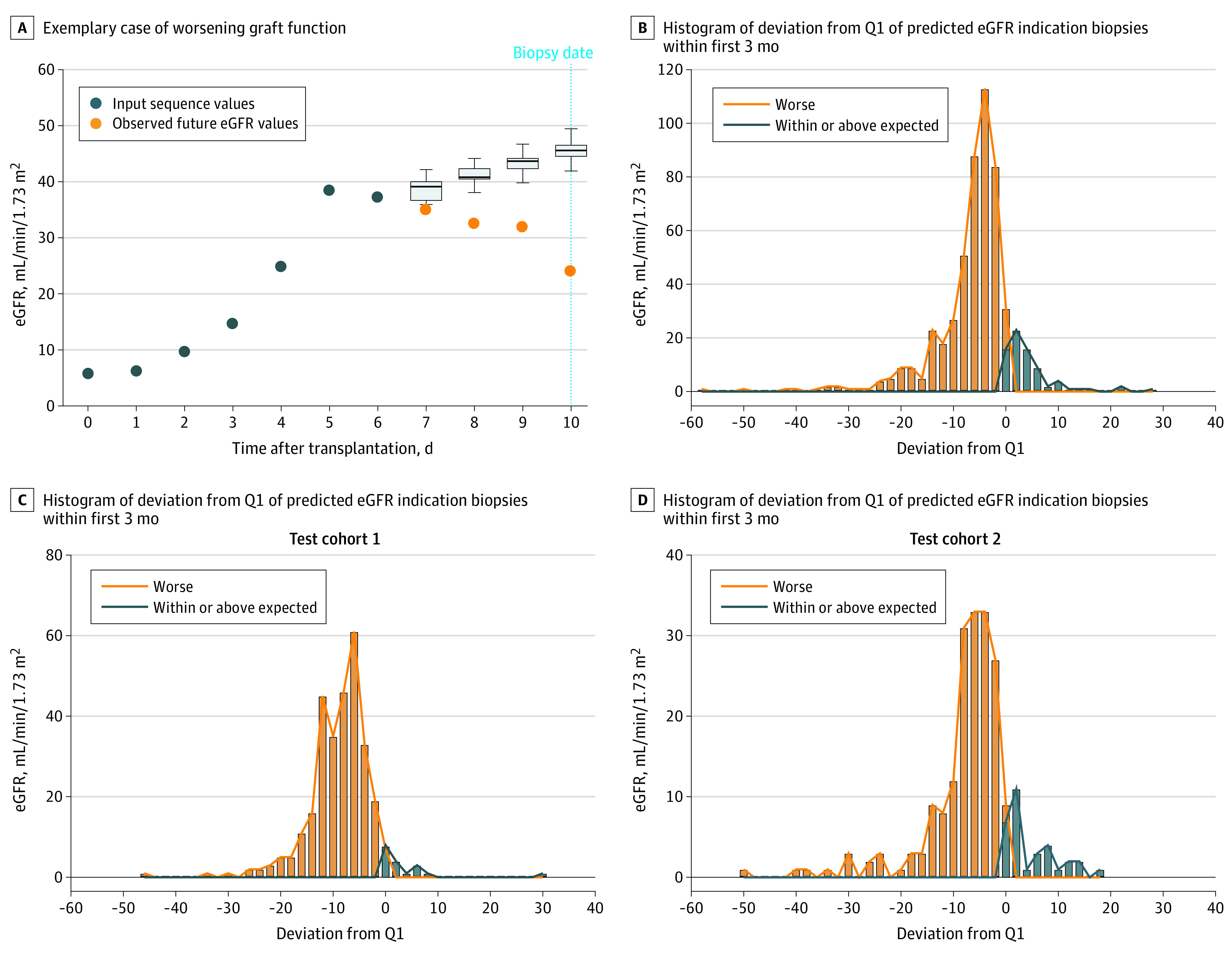
Clinical Performance of the Sequence-to-Sequence Models A, Exemplary case of worsening graft function when comparing the actual eGFR at the time of the biopsy compared with the predicted eGFR from the models (represented by the boxplots). B, Histogram of the deviation of actual eGFR values at the time of an indication biopsy compared with the lower quartile of the predicted eGFR. Samples are classified as worsening graft function when the actual eGFR was below the lower quartile (Q1) of the predicted eGFR by the sequence-to-sequence model. C, Histogram of deviation of actual eGFR values at the time of an indication biopsy for test cohort 1. D, Histogram of deviation of actual eGFR values at the time of an indication biopsy for test cohort 2. eGFR indicates estimated glomerular filtration rate; Q1, quartile 1.

We then assessed whether the sequence-to-sequence model predictions were concordant with the clinical estimation of worsening graft function, as illustrated by performing an indication biopsy. Estimating the deviation between real measured eGFR values and the predicted sequences at time of indication biopsies was thought to provide insight in the sensitivity of the model for clinically relevant graft functional deterioration. Ultimately, such sensitivity is needed for potential use as alarm system in artificial intelligence–based clinical decision support. According to the sequence-to-sequence models, 478 of 555 actual eGFR values at the time of the biopsies fell below the IQR of the predicted eGFR values, which yields a sensitivity of 86.1% (Figure 3A-B). Of the 555 indication biopsies, 233 (42.0%) had evidence of acute rejection assessed according to the Banff 2019 classification,^12^ of which 85.4% (199 of 233) were detected in indication biopsies with actual eGFR below the IQR of the predicted eGFR.

### Independent Validation

Next, we applied the prediction models from the derivation cohort on the 2 independent test cohorts. Similar error rates were found compared with the training cohort (Figure 1). In the test cohorts, we also assessed the deviation in real measured eGFR from the predicted eGFR at the day of a biopsy for graft dysfunction in the first 3 months, using the same strategy as in the training cohort. In the first test cohort, 293 of 311 (94.2%) biopsies had a measured eGFR below the IQR of the predicted eGFR and in the second test cohort, 181 of 213 (85.0%) biopsies had a measured eGFR below the IQR of the predicted eGFR (Figure 3C-D), indicating a worsening graft function against the benchmark predicted range.

### Extension Beyond 3 Months Post Transplant

When we applied this algorithm beyond 3 months post transplant, the errors correlated proportionally to the demanded output lengths, but were less dependent on the input lengths, which started already at a minimum of 180 days of eGFR values post transplant. Overall root mean square errors remained low for output lengths requested up to 1 year (eFigure 4 in the Supplement).

## Discussion

Using recurrent neural network sequence-to-sequence modeling, we developed a tool to forecast patient-specific kidney transplant function. Rather than aiming at prognostication of graft failure using kidney function trajectories, this sequence-to-sequence model demonstrated accurate predictions of the individual recipients’ eGFR values for variable time spans in the first 3 months after transplant based on the patient’s previous eGFR values. The performance was validated in independent cohorts. Overall, this model aids in defining patient-specific expected ranges of graft function, taking into account fluctuations in kidney function associated with biological and technical variability. With this ability, our algorithm can help identify when a new deviating measure can be considered a true anomalous value on a patient-specific level.

The clinical assessment of trajectories of kidney function was previously modeled statistically and evaluated for prognostication, for example by using latent class mixed models^13,14^ and ARIMA,^15,16,17^ but prediction of future patient-specific expected ranges, to benchmark new measured values against, is specific to this study. This model incorporates previous eGFR trajectories in its prediction, with better accuracy than a more conventional ARIMA model. The robustness of the predictions was dependent on the input and output length for the model: the accuracy increased proportionally when a longer sequence of previous eGFR values was given as input and was better for more adjacent than longer-term predictions.

The clinical potential of this model may lie in its ability to provide a patient-specific expected reference range of future eGFR values to which each new observed eGFR value can be benchmarked. This process could then lead to the creation of alarms when a new value is deviating below the expected for this patient. This potential was illustrated by reviewing the deviation between measured and predicted eGFR at the time of biopsies performed for graft dysfunction. Clear deviation from the predicted eGFR values was identified by the algorithm in 85% to 94% of cases. The inherent fluctuating nature of eGFR often creates doubt in clinicians on whether a decrease in eGFR may be considered as still within the reference range of fluctuation or whether the eGFR should alert them to prompt a biopsy. Results of our study suggest the potential for the sequence-to-sequence models in clinical decision support in indicating to clinicians when measured kidney function is deviating from the predicted values, which could prompt clinical action such as performing a kidney transplant biopsy to exclude or diagnose rejection.

To our knowledge, this is the first study to include a deep learning eGFR forecasting model in the field if kidney transplantation. Machine learning algorithm-based forecasting prediction models, such as recurrent neural network have been widely used in other fields outside medicine, especially in the natural language processing domain.^18,19^ Recurrent neural network–based models can have better performance than ARIMA models^20,21,22,23^ and clinical experts.^24^ Beyond the field of kidney transplantation and kidney disease, our sequence-to-sequence models could be tested in other chronic diseases that require systematic laboratory-based monitoring. Current laboratory reporting systems and electronic medical records do not report the patient-specific range but instead report the reference range for the healthy population, which greatly diminishes the value of these reference ranges in the follow-up of chronic conditions.^25^ Therefore, our model could guide patients and caregivers in estimating a true deviation from the expected laboratory values rather than relating the individual results to the reference range of the healthy population.

### Limitations

This study has limitations. First, the model does not take into account other variables than eGFR, such as proteinuria or donor-specific antibody development, which could also prompt a biopsy. Furthermore, the study population consisted largely of White kidney transplant recipients, limiting generalizability to other populations. The full eGFR history per patient was taken into account, meaning that patients with multiple indication biopsies and delayed graft function were also included. This inclusion could have negatively affected the accuracy of the sequence-to-sequence predictions in some cases. In addition, this model does not take into account histologic information and might predict graft functional decline in slowly progressive pathologic processes, which might then be considered as graft functional decline in the expected range of normal, whereas histologic injury might be present. These chronic progressive processes could limit extension of this model beyond 1 year, when more chronic lesions such as interstitial fibrosis and tubular atrophy can cause a gradual functional decline. The clinical potential was retrospectively assessed; further prospective validation in clinical practice is needed. Limitations are inherent to the formulas for estimating GFR,^26,27^ and even when using serum creatinine, there is intrinsic error in the measurement.^28^ Therefore, we cannot expect complete accuracy of the predictions. Nonetheless, the value of the model may lie in defining a relative range of intra-individual variation rather than snapshot absolute values.

## Conclusions

In this diagnostic study, our sequence-to-sequence model was able to forecast patient-specific kidney function after transplant. The first months posttransplant are marked by great fluctuations in kidney function, which may create alarm in clinicians and uncertainty in patient. The increasing use of electronic medical records linked to laboratory information systems could facilitate implementation of the model into the health information technology infrastructure and software used to care for patients. The implemented model could automatically indicate the projected expected range of eGFR values per patient, compare this range with new measured eGFR values to assess deviations, which would lead to generation of alarms and incorporate each new eGFR measurement to update the predictions automatically. Our prediction model could guide clinicians in whether eGFR fluctuations should be considered to be alerts or rather expected for the specific patients instead of relating the individual results to the reference range of the healthy population. The clinical utility of this prediction model in decision-making in addition to current interpretation of eGFR trajectories by experienced clinicians may need to be evaluated in prospective clinical trials.

## Notes

Supplement. **eMethods.** Glossary, Model, Background, and Parameters **eFigure 1.** Representation of Missing Values of eGFR in the First 3 Months After Transplantation **eFigure 2.** Development and Cross-Validation of the GRU Seq2Seq Models **eFigure 3.** Intra-and Inter-Individual Variability of eGFR Trajectories **eFigure 4.** Robustness of the Model for Long-term Extension Beyond 3 Months Post-Transplantation in the Derivation Cohort, as Illustrated by Mean RMSE per Given Input and Demanded Output Length
