# Supplementary material for: Forecasting of Patient-Specific Kidney Transplant Function With a Sequence-to-Sequence Deep Learning Model
Source: JAMA Netw Open. 2021 Dec 30;4(12):e2141617. doi: 10.1001/jamanetworkopen.2021.41617 (PMC8719239; doi:10.1001/jamanetworkopen.2021.41617)
Supplement: Supplement. — eMethods. Glossary, Model, Background, and Parameters eFigure 1. Representation of Missing Values of eGFR in the First 3 Months After Transplantation eFigure 2. Development and Cross-Validation of the GRU Seq2Seq Models eFigure 3. Intra-and Inter-Individual Variability of eGFR Trajectories eFigure 4. Robustness of the Model for Long-term Extension Beyond 3 Months Post-Transplantation in the Derivation Cohort, as Illustrated by Mean RMSE per Given Input and Demanded Output Length [file jamanetwopen-e2141617-s001.pdf]

Supplemental Online Content

Van Loon E, Zhang W, Coemans M, et al. Forecasting of patient-specific kidney transplant function with a sequence-to-sequence deep learning model. *JAMA Netw Open.* 2021;4(12):e2141617. doi:10.1001/jamanetworkopen.2021.41617

eMethods. Glossary, Model, Background, and Parameters

eFigure 1. Representation of Missing Values of eGFR in the First 3 Months After Transplantation

eFigure 2. Development and Cross-Validation of the GRU Seq2Seq Models

eFigure 3. Intra-and Inter-Individual Variability of eGFR Trajectories

eFigure 4. Robustness of the Model for Long-term Extension Beyond 3 Months Post-Transplantation in the Derivation Cohort, as Illustrated by Mean RMSE per Given Input and Demanded Output Length

This supplemental material has been provided by the authors to give readers additional information about their work.

© 2021 Van Loon E et al. *JAMA Network Open.*

eMethods. Glossary, Model, Background, and Parameters

Glossary

- Seq2Seq model: a recurrent neural network (RNN) based model with an encoder-decoder architecture, which takes a sequence as input and outputs a sequence.
- GRU (Gated Recurrent Unit): is a part of a specific model of recurrent neural network, which uses a gating mechanism to allow RNNs to learn the long-term dependency efficiently.
- RMSE (Root mean square error): measures the error between values predicted by a model and the values observed.
- k-fold cross validation: a resampling procedure used to evaluate the performance of machine learning models on a limited data set. In k-fold cross validation, the data set is split into k subsets of equal size, where in each subset the model is validated by applying the model that is trained in k-1 subsets1.
- batch size: the number of samples that will be propagated through the network in one iteration. As the training data become larger in longer term analysis, using a smaller batch size requires a significant smaller memory use2.

ARIMA model

An ARIMA model is a generalization of Autoregressive Moving Average (ARMA), which combines Autoregressive (AR) and Moving Average (MA) processes. An ARIMA model (p,d,q) captures the following key elements of the model:

- AR: Autoregression. It is a regression model that uses the dependencies between an observation and a number of lagged observations (p).
- I: Integrated. It is used to make the time series stationary by measuring the differences of observations at different times (d).
- MA: Moving Average. It is an approach that takes the dependency between observations and the residual error terms into account, when a moving average model is applied to the lagged observations (q).

The ARIMA model applies the differencing process d times (a parameter for the differencing order) to handle the non-stationary data. We did the Augmented Dickey–Fuller (ADF) test on the derivation cohort (first 3 months after transplantation) to check the stationarity of each time series. In total, 70% of patients had stationary egfr trajectories. In order to find the optimal model for this dataset, we applied a grid search optimization method to decide the parameters of the ARIMA model, including differencing order d. Firstly, we gave a range of values between 0 and 5 for all parameters. Then the grid search performs exhaustive searching through this subset of the model parameter space. In other words, all possible parameter combinations in that specified range are considered and evaluated. Each combination of parameters is fit to the model and evaluated by RMSE (Root Mean Square Error) values. The final model was chosen with the lowest RMSE. Additionally, the range of (0, 5) was selected due to the increasing RMSE beyond 5. Based on results from grid search, the optimized p, d and q values are: p = 1, d = 0 and p = 0. Differencing order d is selected as 0, which means no differencing needs to be done to this data. This also supports the ADF test result that most of the data are stationary.

Background on RNN, LSTM and GRU and the choice for the Seq2Seq model

1) Recurrent Neural Networks and Gated Recurrent Unit:

Recurrent neural networks(49) are a variety of conventional feedforward neural networks, which are able to model long-term dependencies by its self-loop connections and shared parameters across various time steps. Given a sequence x = (x_1,x_2,….x_T), RNN update their recurrent hidden state h_t, formally by:

$$h_t = \begin{cases} 0, t = 0 \\ \emptyset\ (h_{t-1}, x_t), \text{otherwise} \end{cases}$$

Where ∅ is a nonlinear activation function such as a logistic sigmoid with an affine transformation. Traditionally, a standard RNN computes a sequence of outputs (y_1,y_2,….y_T) by iterating the following equations(50):

$$h_t = g\left(W^{hx}x_t + W^{hh}h_{t-1}\right)$$
$$y_t = W^{yh}h_t$$

However, many studies have reported the vanishing or exploding gradients problem when using RNNs modelling due to multiplicative gradient exponentially decreasing or increasing in terms of the number of

© 2021 Van Loon E et al. *JAMA Network Open.*

layers(50). Therefore, a Long Short Term Memory (LSTM) unit(19) was developed to solve this problem and this became the most effective method for handling long sequences. LSTM is a more sophisticated activation function with affine transformation followed by a simple element-wise nonlinearity by using gating units. Essentially, LSTM is capable of deciding which information should be remembered or forgotten by the gates. Intuitively, LSTM captures the important features from the input sequence at each stage and remembers this information over a long distance(50).
Recently, a similar model was proposed, Gated Recurrent Unit (GRU)(20) with a more simple structure than LSTM and more efficient computations(51). Therefore, GRU was chosen for this study. GRU is defined by following equations:

$$r_t^j = \sigma(W_r x_t + U_r h_{t-1})^j \quad (1)$$
$$z_t^j = \sigma(W_z x_t + U_z h_{t-1})^j \quad (2)$$
$$\tilde{h}_t^j = \tanh(W x_t + U(r_t \odot h_{t-1}))^j \ (3)$$
$$h_t^j = (1 - z_t^j) h_{t-1}^j + z_t^j \tilde{h}_t^j \ (4)$$

The most characteristic element of the GRU is (4), where the activation h_t^j of the GRU at time t is a linear interpolate between the previous activation h_{t-1}^j and the candidate activation$\tilde{h}_t^j$. The update gate z_t^j modulates the interpolation by deciding how much the unit updates its activation (2). The candidate activation $\tilde{h}_t^j$ is computed similarly to a standard recurrent unit update (3), but includes an additional modulation of a reset gate r_t^j(1)(50, 52).
2) Sequence to sequence model:
Unlike previously developed RNN-based multi-step prediction models, which output only one value per prediction, sequence to sequence (Seq2Seq) models can generate a sequence directly. More importantly, RNN have a significant limitation, requiring the input and output lengths to be known and fixed. Original Seq2Seq model used LSTM to learn to map input sequences with various lengths into a fixed-dimensional vector representation(52). In this work, however, we used GRU to build the model.
Preprocessing
eGFR values were scaled from 0 to 1 as a preprocessing normalization step.
Encoder-decoder concept:
The Seq2Seq model uses the encoder-decoder concept, where the recurrent encoder encodes the input sequence vector and the decoder uses it for the output sequence generation.
Seq2Seq models aim to map an input sequence of vectors X = (x_1,…,x_N) to the output sequence Y = ($y_{1,...}y_M$). Formally, the encoder reads the input sequence and each RNN cell (i.e. GRU) computes[29] as

$$h_t = f(x_t, h_{t-1})$$

where x_t is the current input, and h_{t-1} , h_t are the previous and the current cell's hidden states, respectively. Then the encoded vector (representation of the input) ***c*** is calculated by using all hidden layers of cells. Here we followed the setting from Sutskever et al.[15]:

$$\boldsymbol{c} = q(h_1, \dots, h_N) = h_N$$

Then GRU use the obtained encoded vector ***c*** to estimate the conditional probability $p(y_1, \dots, y_M | x_1, \dots, x_N)$ by the following equation:

$$p(y_1, \dots, y_M | x_1, \dots, x_N) = \prod_{t=1}^{M} p(y_t | \boldsymbol{c}, y_1, \dots, y_{t-1})$$

Let us denote each input time series per patient as X = (x1, x2,...,xt), which refers to eGFR measurements from day 1 after transplantation to day t. Then the output time series is denoted as Y = (yt+1,yt+2,..., yt+n), which refers to predicted eGFR values from day t+1 to day t+n. Our proposed Seq2Seq model then maps X to Y directly. During the whole development of the model, the eGFR values from the future were never used to forecast values in the past.
Construction of models with different input and output length
By training 126 models with each a different input and output length, we next evaluated how the forecasting performance evolved with different length of in- and output. Each model with pre-specified in- and output consisted of 5-fold trained models, each contributing to a prediction in the forecasting phase and yielding a measure of uncertainty in the predictions1. We translated this uncertainty measure into an interquartile range (represented in a boxplot) for each prediction.

© 2021 Van Loon E et al. *JAMA Network Open.*

Hyperparameters of the GRU Seq2Seq model
After intensive empirical optimization, we determined the hyperparameters of the GRU Seq2Seq model:

- Number of hidden layers in encoder and decoder: 2, 2
- Number of GRU cell per layer in encoder and decoder: 64, 64
- Optimizer: Adam
- Learning rate: 0.007
- Activation function: RELU
- L2 Regularizer: 0.000001
- Batch size(2): 256(2); 32(for long term)
- Epoch: 500
- Cross_validation: 5 folds
- Early stopping patience: 20
- Loss: mean squared error
- Inner validation data: 20% training data
-

Validation process
First, the GRU Seq2Seq models were cross-validated on the derivation cohort and locked. For the internal validation, we utilized 5-folds cross validation for splitting the total number of patients into a training dataset (80% patients) and testing dataset (20% patients). Before evaluating the performance, the predictions underwent post-processing including removal of imputed eGFR values and inversion of the normalization, in order to adequately compare with the observed eGFR values. The performance of the models was evaluated using RMSE for each-fold-trained model between each predicted eGFR value from the output sequence and the real measured eGFR on the same day. The final Seq2Seq model performance was obtained by averaging the results of those 5-fold-trained models. In the second step, the 5 trained and locked models were applied to the independent test cohorts. Each forecasting sequence contained 5 candidates (from 5-fold-trained models), which were used to calculate the mean and median of the candidate sequence predictions and for subsequent RMSE estimations.
Extension beyond 3 months
For testing the accuracy of the GRU Seq2Seq model beyond 3 months post-transplant, we trained new models for longer term input and output sequences of the derivation cohort, using a smaller batch size (m= 32, compared to m= 256 for the 3 months model), but with unaltered model architecture, again using 5-fold cross-validation. Similar to the short-term input and output length models, model accuracy beyond 3 months was assessed by calculating RMSE between each predicted eGFR value from the output sequence and the real measured eGFR on the same day.

© 2021 Van Loon E et al. *JAMA Network Open.*

eFigure 1. Representation of missing values of eGFR in the first 3 months after transplantation. The boxplots represent the largest number of missing eGFR values between two observations for all patients in the first 3 months after transplantation. For the application of the model, the missing values were imputed using duplication, meaning the last observation was carried forward in the definition of the input eGFR sequences.

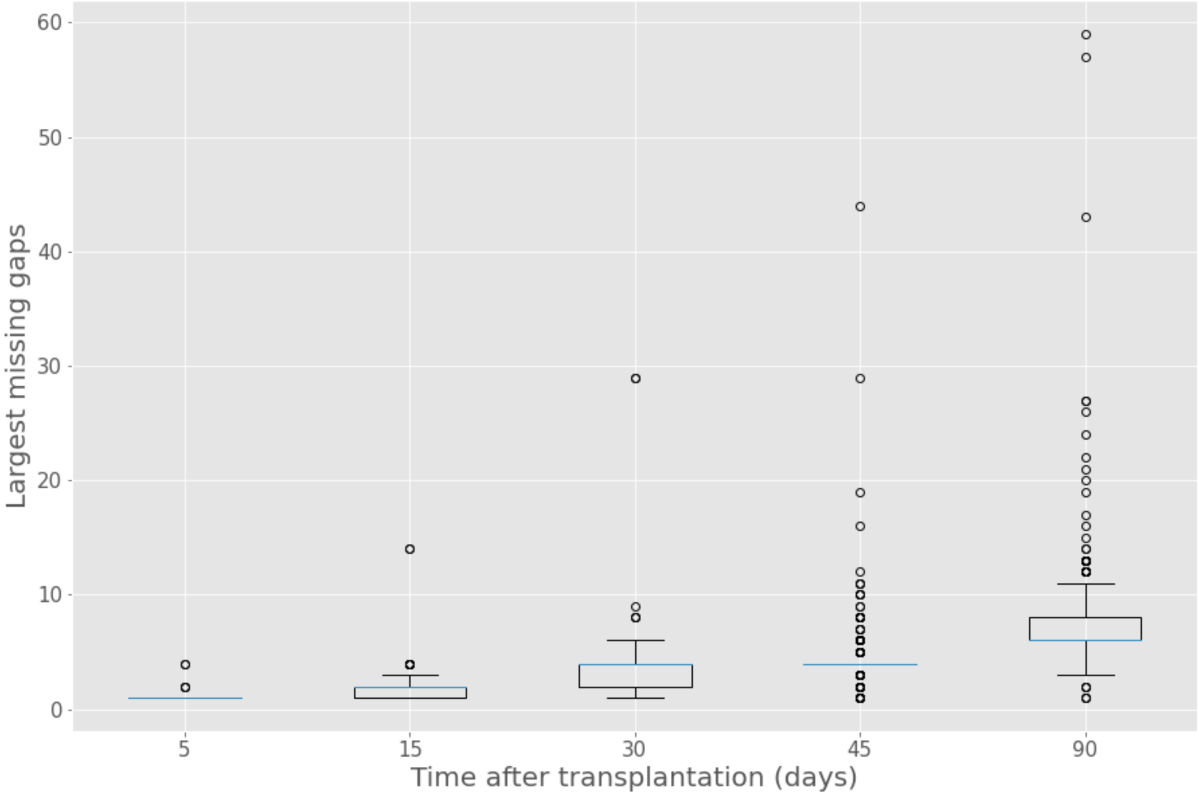

© 2021 Van Loon E et al. *JAMA Network Open.*

eFigure 2. Panel A. Development of the GRU Seq2Seq models for forecasting patient-specific kidney transplant function. The top panel shows the pipeline of the proposed model. The lower panel schematically illustrates the sequence-to-sequence model encoder-decoder architecture. Let us denote each input time series per patient as X = (x1, x2,...,xt), which refers to eGFR measurements from day 1 after transplantation to day t. Then the output time series is denoted as Y = (yt+1,yt+2,..., yt+n), which refers to predicted eGFR values from day t+1 to day t+n. Our proposed Seq2Seq model then maps X to Y directly. During the whole development of the model, the eGFR values from the future were never used to forecast values in the past. First, N different patients' (P1 to PN) continuous eGFR values (input sequence values, X1– XT, blue crosses) were pre-processed and then analyzed by the sequence-to-sequence model. The predictions from the model (Y1–YT', green crosses) were post-processed and then evaluated by comparison with the observed eGFR values (red crosses) using the root mean square error (RMSE). GRU, gated recurrent unit. eGFR: estimated glomerular filtration rate. Panel B. Cross-validation and independent validation of the GRU Seq2Seq models. The validation process consisted of two steps. In Step 1, the model was built on the derivation cohort using 5-fold cross-validation for splitting the total number of patients into a training dataset (80% patients) and testing dataset (20% patients) to fine-tune the parameters and evaluate model performance. After 5-fold-trained models were made, we computed the performance via RMSE for each fold-trained model ("i"). The final Seq2Seq model performance was obtained by averaging the results of those 5-fold-trained models. In Step 2, the models were tested on independent test cohorts ('Test cohorts') with the 5 trained and locked models, without modification. Each forecasting sequence contained 5 candidates (from 5-fold-trained models), which were used to calculate the mean and median of the candidate sequence predictions and for subsequent RMSE estimations. RMSE, root mean square error.

© 2021 Van Loon E et al. *JAMA Network Open.*

A.

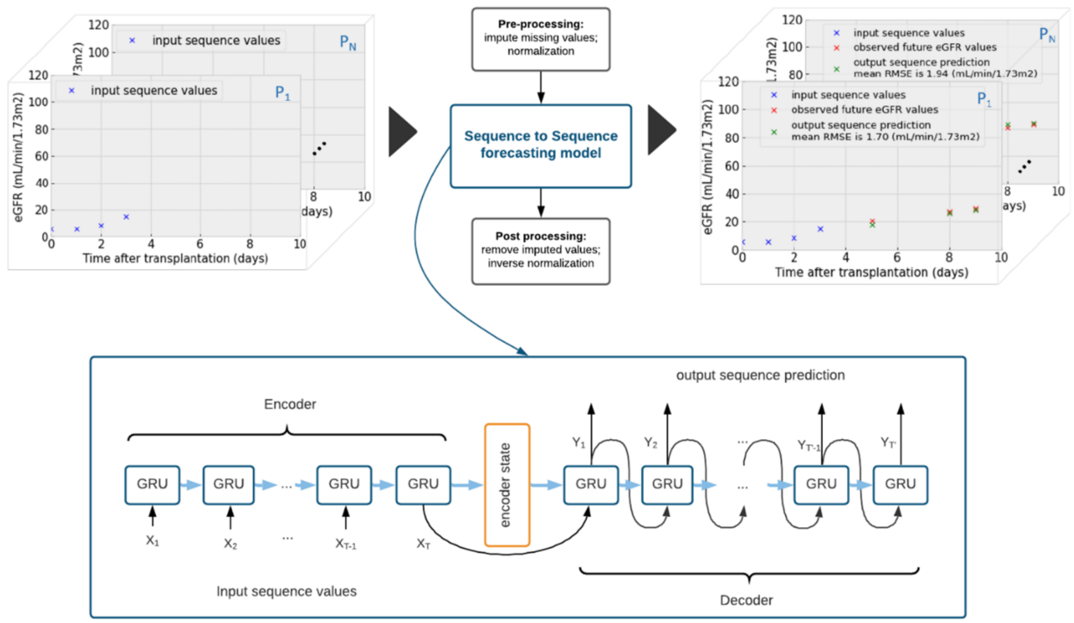

B.

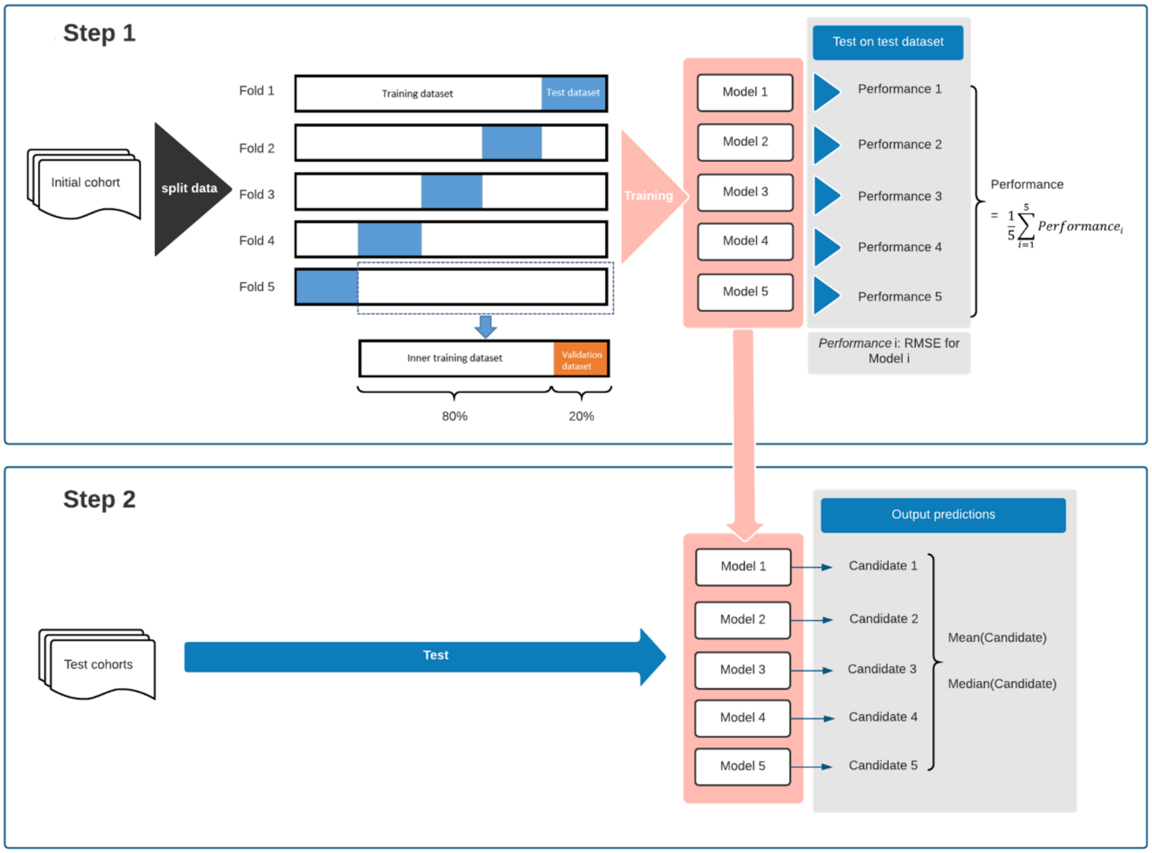

© 2021 Van Loon E et al. *JAMA Network Open.*

eFigure 3. Intra-and inter-individual variability of eGFR trajectories. **a.** Spaghetti plot of randomly selected 100 patients' eGFR values in the derivation cohort over time until end of follow-up or graft failure. **b.** Daily percentage change (using linear interpolation for the days without eGFR values) for all patients of the derivation cohort until end of follow-up or graft failure. eGFR: estimated glomerular filtration rate.

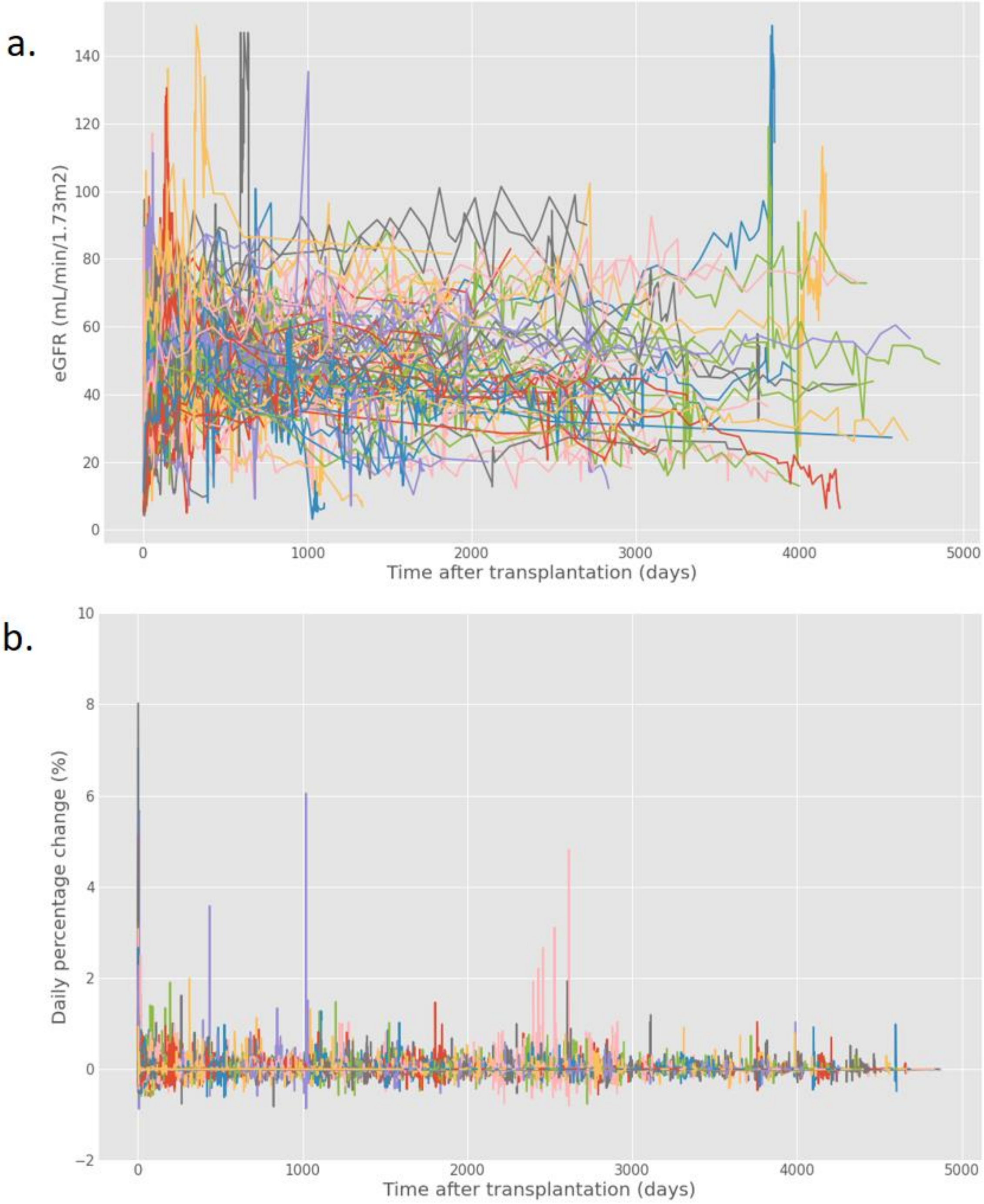

© 2021 Van Loon E et al. *JAMA Network Open.*

eFigure 4. Robustness of the model for long-term extension beyond 3 months post-transplantation in the derivation cohort, as illustrated by mean RMSE per given input and demanded output length. RMSE, root mean square error.

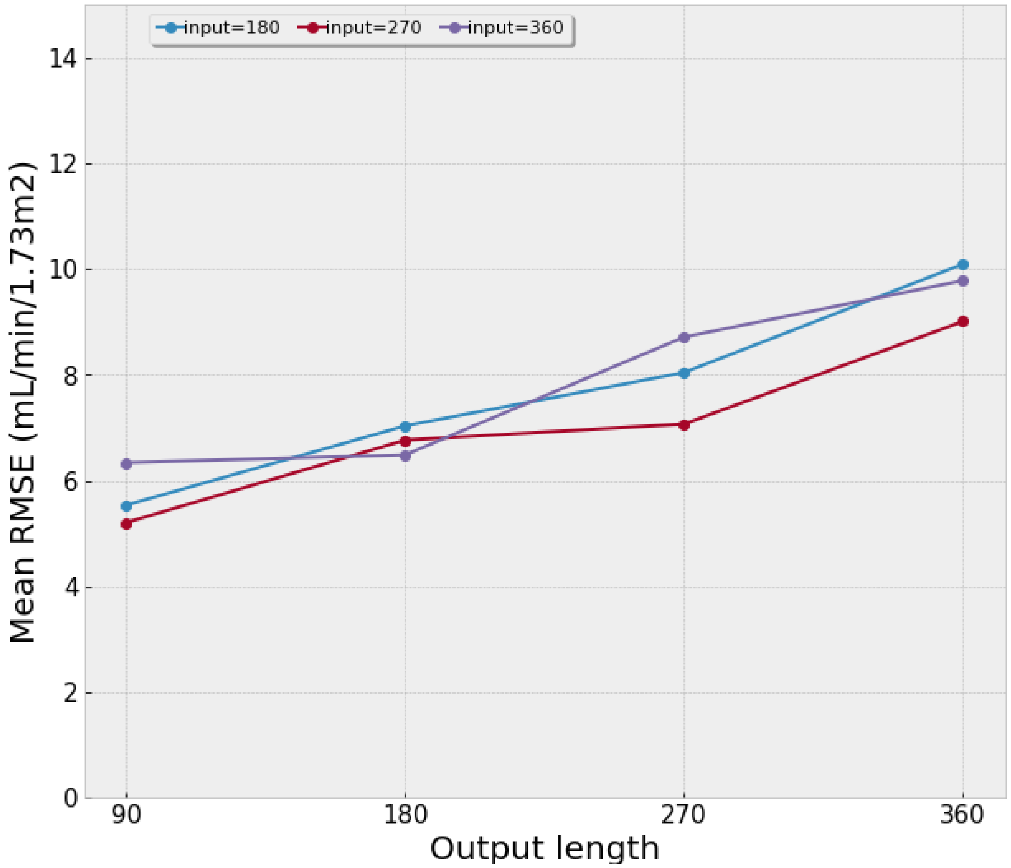

© 2021 Van Loon E et al. *JAMA Network Open.*
